# Acute Effects of Intermittent Foot Cooling on 1 RM Leg Press Strength in Resistance-Trained Men: A Pilot Study

**DOI:** 10.3390/ijerph18189594

**Published:** 2021-09-12

**Authors:** Chih-Min Wu, Mei-Hsien Lee, Wen-Yi Wang, Zong-Yan Cai

**Affiliations:** 1 Department of Leisure and Sports Management, Cheng Shiu University, Kaohsiung 83300, Taiwan; sugicalwu@gmail.com; 2 Department of Mathematics, University of Taipei, Taipei 100234, Taiwan; meihsien@go.utaipei.edu.tw; 3 Graduate Institute of Sports Pedagogy, University of Taipei, Taipei 111036, Taiwan; wenyiwang1225@gmail.com; 4 Center for Physical and Health Education, SiWan College, National Sun Yat-Sen University, Kaohsiung 804201, Taiwan

**Keywords:** electromyography, arousal, muscle strength, immersion, quadriceps muscle

## Abstract

Inter-set peripheral cooling can improve high-intensity resistance exercise performance. However, whether foot cooling (FC) would increase 1 repetition maximum (RM) lower-limb strength is unclear. This study investigated the effect of intermittent FC on 1 RM leg press strength. Ten recreational male lifters performed three attempts of 1 RM leg press with FC or non-cooling (NC) in a repeated-measures crossover design separated by 5 days. FC was applied by foot immersion in 10 °C water for 2.5 min before each attempt. During the 1 RM test, various physiological measures were recorded. The results showed that FC elicited higher 1 RM leg press strength (Δ [95% CI]; Cohen’s d effect size [ES]; 13.6 [7.6–19.5] kg; ES = 1.631) and electromyography values in vastus lateralis (57.7 [8.1–107.4] μV; ES = 0.831) and gastrocnemius (15.1 [−3.1–33.2] μV; ES = 0.593) than in NC. Higher arousal levels (felt arousal scale) were found in FC (0.6 [0.1–1.2]; ES = 0.457) than in NC. In conclusion, the preliminary findings, although limited, suggest intermittent FC has a potential ergogenic role for recreational athletes to enhance maximal lower-limb strength and may partly benefit strength-based competition events.

## 1. Introduction

Lower-limb muscle strength is crucial in strength and power sports [1]. For instance, individuals who possess greater lower-limb muscle strength are often capable of eliciting superior power abilities, including various jumping capacities [2,3], acceleration [4], and sprint time [3,4]. Collectively, these may facilitate broad aspects of sports performance. As reported by Golas et al. [5], these include most team sports, combat sports, as well as track and field sprints, throws, and jumps. Furthermore, greater lower-limb strength is necessary for competitive Olympic weightlifters and powerlifters [6]. Therefore, exploring accessible strategies for effectively enhancing maximal lower-limb muscle strength may be of great value.

Peripheral cooling strategies have recently attracted attention given their promising effects on neuromuscular activation [7,8,9] and resistance-based exercise performance [10,11,12,13,14]. That is, cooling the joints near exercising muscles, [7,8,9] or the extremities (e.g., palms [10,11,12,13] or feet [14]) far from exercising muscles rather than cooling the muscle itself may attenuate performance because of reduced muscle temperature below the optimal range [15,16]. Cooling the ankle increases motor neuron excitability as assessed by artificial electrical stimulation [7]. Resistance exercise studies demonstrated that palm cooling between a four-set, eight-repetition concentric flywheel leg press at a submaximal level of effort hastens blood lactate clearance and delays average power decrements [11]. In addition, during four sets of 85% one-repetition maximum (1 RM) bench press to exhaustion workout, inter-set palm cooling at 10 °C for 2.5 min increases the repetitions, total exercise volume, and root mean square (RMS) electromyography (EMG) of some exercising muscles for resistance-trained male and female participants [12,13]. Another recent study further explored the effect of inter-set foot cooling (FC) on leg press pyramid workouts. The investigators instructed the resistance-trained participants to perform four sets of 85–90% 1 RM leg press exercise to exhaustion with inter-set FC or non-cooling (NC). Results showed that a leg press workout performed with inter-set FC increases the number of repetitions completed per set and the RMS EMG of the vastus lateralis (VL) without increasing the degree of rating of perceived exertion (RPE), thus reinforcing the notion that cooling the peripheral extremities is advantageous for strength performance [14]. The exact mechanism by which peripheral cooling enhances repeated resistance-based exercise performance is yet to be fully elucidated, but proposed factors include activation of peripheral afferent stimulation, excitation of the central nervous system (CNS) arousal level, facilitation of motor neuron pool excitability [7,8,9,12,13,14], and delay of fatigue [10,11,12,13]. However, arousal level indicators have not been examined in resistance exercise research.

The numbers of repetition > 85% 1 RM leg press exercise have been improved by FC, thus indicating that the hypothetical 1 RM strength may also improve from prediction equations [17]. There is evidence that 20 min cooling using ice bags applied to the knee joint increases knee extension maximal voluntary contraction (MVC) [9]. However, the single-joint exercise of knee extension in real competition or training scenarios less closely mimics sports-specific movement patterns, and such a long rest interval for 20 min between bouts is usually not feasible. Whether or not a short period of 2.5 min of peripheral cooling as used elsewhere in resistance exercise workouts can benefit the maximal effort of an actual multiple-joint 1 RM attempt is unclear. Moreover, studies should confirm whether or not the increase in the number of repetitions to exhaustion during repeated high-intensity resistance exercise workouts is derived from the substantial increase in maximum strength or if it may be due to the anti-fatigue effect that increases muscle endurance at the submaximal effort.

Accordingly, this study primarily aimed to investigate the effects of intermittent FC on 1 RM leg press strength. Moreover, during the 1 RM test, strength performance-related physiological variables including sole skin temperature, arousal level, EMG, and RPE response were measured as the secondary outcomes. We hypothesized that intermittent FC enhances maximal strength, elevates arousal level, and amplifies EMG response during a 1 RM leg press test.

## 2. Materials and Methods

### 2.1. Study Design

**Figure 1:**
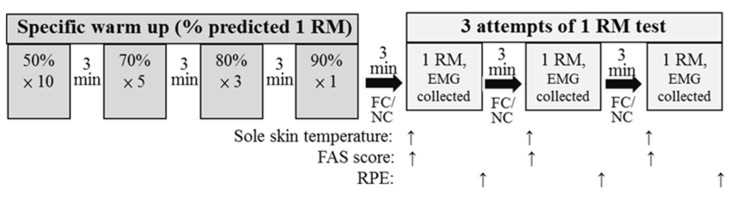
Schematic of the experimental design. Procedures for warm up and three attempts of 1 RM test in FC and NC conditions, including a schedule of parameters collected. RM—repetition maximum; FAS—felt arousal scale; EMG—electromyography; RPE—rating of perceived exertion; FC—foot cooling; NC—non-cooling.

One familiarization day followed by two 1 RM leg press test days with or without intermittent FC in a randomized, balanced, within-group research design was performed (Figure 1). The 2 test days were separated by 5 day intervals. The familiarization day entailed familiarization with study procedures, collection of individuals’ anthropometric data, and familiarization with the leg press equipment. During the 1 RM leg press test days, participants performed three attempts with intermittent FC or NC following a standardized warm-up protocol. The dominant lower-limb EMG data during the concentric phase of the 1 RM leg press exercise and sole skin temperature, arousal levels, and RPE values during the workouts were obtained. All experiments were performed in a normothermic room, which was maintained at 24–26 °C and 40–60% humidity, and at the same time of day for each participant. All experimental days were conducted under the strict, direct supervision of at least two experienced researchers familiar with technical resistance training.

### 2.2. Participants

Eligible participants were volunteers who met the inclusion criteria and gave their consent to participate in the study. As mandatory requirements, participants were over 20 years of age, had participated in regular weight training for a minimum of six months, and classified as healthy individuals with no major medical history. Participants were excluded if they had a history of lower body injury in the previous 6 months, and reported taking ergogenic supplements or medicine that could affect their exercise performance. A total of 10 healthy men included in this study (age: 21.5 ± 0.8 years, stature: 175.5 ± 6.8 cm, body mass: 76.8 ± 15.6 kg, training experience: 1.7 ± 1.2 years). The participants were instructed to maintain their usual diets during the study but to refrain from strenuous exercise, caffeine, and alcohol 24 h before each trial and a pre-workout meal 1–3 h before each condition [11]. In addition, participants were instructed to cease performing resistance-training exercises during the study period. The sample size was calculated using G*Power software, version 3 (Franz Faul, Christian-Albrechts -Universität Kiel, Kiel, Germany), using the date from a previous study that investigated the effects of FC on repeated high-intensity leg press working volume [14]. To detect statistically significant improvements in 1 RM leg press strength with a power of 80%, an alpha level of 5% and an effect size (ES) of 1.259 from previous study [14], a minimum of 6 participants was required. The participants were fully informed of the experimental procedure, potential risks and benefits of the study and signed informed consent documents before their participation. The study was conducted according to the guidelines of the Declaration of Helsinki, and approved by the National Cheng Kung University Human Research Ethics Commite (NCKU HREC-E-108-080-2).

### 2.3. Intervention

The cooling method with cold water immersion has been applied in many studies [14,18,19]. In the present study, the participants immersed their feet up to the distal end of the fibula (lateral malleolus) in buckets filled with water controlled at 10 °C ± 1 °C as previously described [14]. The water temperature throughout the FC condition was monitored, and the target temperature was maintained by adding crushed ice when needed [14,19]. Benefits in the bench press workout were observed after application of this temperature in palm cooling [12,13], and an ergogenic effect on the leg press pyramid workout was found when using foot immersion [14]. For the FC condition, they water-cooled their feet for 2.5 min before the first attempt and between each attempt of maximal strength lifting (Figure 1). The NC condition involved placing the feet in the buckets but without application of cold water.

### 2.4. Outcome Measures

#### 2.4.1. RM Test

The 1 RM strength was assessed on a resistance machine (Leg Press G3-S70, Matrix, Taipei, Taiwan). The participants were instructed to adjust the seat carriage to set the knee angle at 90° (measured via goniometer). We marked the positions of the feet on the pedal to ensure identical step distance during all tests. Subsequently, the participants squeezed the grips and pushed away the given load until their legs were straight while their head, shoulders, back, and buttocks remained in contact with the pad.

The 1 RM strength test was determined similar to that in relevant studies [12,13,14,20]. A general warm up before the test, consisting of light cardiovascular exercise lasting approximately 5 min, was performed. The test was preceded by specific standardized warm-up sets that included 10 repetitions at 50% of (predicted) 1 RM, 5 repetitions at 70% of 1 RM, 3 repetitions at 80% of 1 RM, and 1 repetition at 90% of 1 RM, followed by 3 attempts to determine the participant’s actual 1 RM. The three attempts were given to mimic a real strength-based competition scenario (powerlifting and Olympic weightlifting). The rest period was 3 min between attempts (Figure 1). The smallest increment between attempts was 0.5 kg.

#### 2.4.2. Sole Skin Temperature

The 3 min rest periods before and between attempts consisted of a 15 s transition from the exercise to treatment, 2 min 30 s of FC or NC condition, and another 15 s transition from the rest period to the next attempt. Sole skin temperature was measured when the participant was ready to begin another attempt. An infrared skin thermometer (DT-8380, Deryuan, New Taipei, Taiwan) was used to measure sole skin temperature. The probe of the thermometer was positioned 0.5 cm from the skin, and the temperature was shown for 1 s on the thermometer display.

#### 2.4.3. Felt Arousal Scale

The felt arousal scale (FAS) was used as a measure of arousal [21]. Immediately before the beginning of each attempt of the 1 RM test workout, participants were instructed to provide their perceived activation by using a poster containing the six-point FAS [21]. Verbal anchors were identified with high scores described by states such as excitement, and with low scores described by states such as relaxation.

#### 2.4.4. Electromyography Signal Analysis

The surface electrodes were attached on the belly of the VL, gastrocnemius (GS), and biceps femoris (BF) muscles of the dominant leg, and the ground electrode was placed over the epicondyle of the tibia after shaving, abrading, and cleaning the participant’s skin with alcohol to minimize impedance. The electrode placement was marked for the following assessments. The participants were instructed not to remove these marks until the 3 experiment days were completed. A Noraxon Telemyo DTS EMG system (Noraxon Inc., Scottsdale, AZ, USA) collected, amplified (×1000), and bandpass-filtered the EMG signals at 10–500 Hz cut-off (Butterworth/Bessels; an antialiasing low-pass filter). EMG data during the concentric phase of the 1 RM leg press exercise were analyzed. To evaluate the motor unit requirements, raw EMG signals were processed as RMS values and were full-wave rectified and low-pass filtered (12 Hz) over the given periods.

#### 2.4.5. Rating of Perceived Exertion

Immediately after each attempt of the 1 RM test workout, the participants provided their RPE value using the Borg CR-10 scale, where 0 indicates extremely easy, and 10 indicates extremely difficult.

### 2.5. Statistical Analysis

The data were expressed as the mean ± standard deviation (SD). A paired *t*-test was used to quantify the differences in the 1 RM and concentric RMS EMG of the 1 RM between conditions. A two-way repeated-measures analysis of variance test was used to quantify the differences in sole skin temperature, arousal score, and RPE values. Significant condition × attempt interactions were followed up with simple main effects analyses, and in cases without significant interaction, the least significant difference (LSD) post hoc tests were used to follow up the significant main effects for each condition and attempt. Cohen’s d was used as an ES measurement for the comparison between two means and interpreted as small (0.2), medium (0.5), large (0.8), and very large (1.30) [22]. Statistical significance was set at *p* < 0.05. Statistical analyses were performed using SPSS 25.0 software (IBM, Armonk, NY, USA).

## 3. Results

### 3.1. Primary Outcome

#### 1 RM Leg Press Strength

A paired *t*-test showed that the heaviest weight lifted for all three attempts in the FC condition was significantly higher than that in the NC condition (*t* = 5.158, *p* < 0.001, Δ [95% CI]; Cohen’s d ES; 13.6 [7.6–19.5] kg; ES = 1.631) (Table 1).

### 3.2. Secondary Outcomes

#### 3.2.1. Sole Skin Temperature

No condition × set interaction (*F* = 2.827, *p* = 0.085, ES = 0.239) and no main effect of set (*F* = 2.834, *p* = 0.086, ES = 0.239) were observed on sole skin temperature. However, a significant main effect of condition (*F* = 465.071, *p* < 0.001, 8.4 [7.5–9.3] °C; ES = 0.981) was identified, with sole skin temperature in FC condition being lower than that in NC condition (1st = 22.3 °C ± 1.6 °C vs. 29.2 °C ± 1.6 °C, 7.6 [6.2–9] °C; 2nd = 21.6 °C ± 1.4 °C vs. 30.0 °C ± 1.4 °C, 8.4 [7.4–9.5] °C; 3rd = 20.9 °C ± 1.9 °C vs. 30.0 °C ± 1.3 °C, 9.1 [8–10.2] °C).

#### 3.2.2. Arousal Score

**Figure 2:**
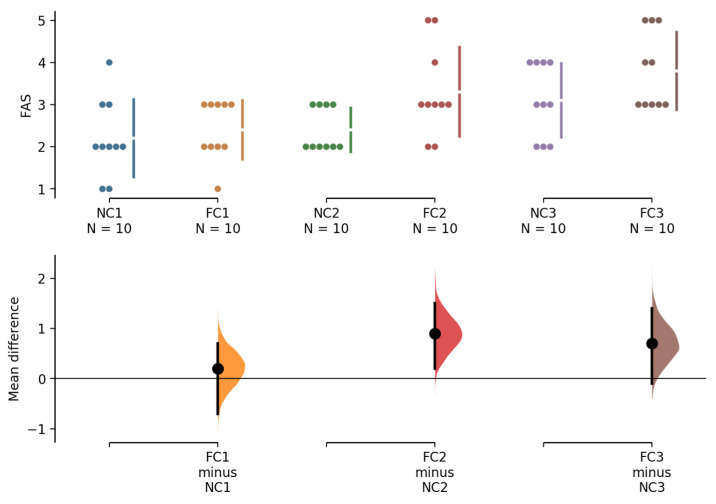
FAS scores prior to attempts of 1 RM leg press test in FC and NC conditions. Gardner–Altman estimation plots showing mean difference. The 95% CI is indicated by the vertical error bar. FAS—felt arousal scale; RM—repetition maximal; FC—foot cooling; NC—non-cooling; CI—confidence interval.

Figure 2 shows the arousal level between conditions. No condition × attempt interaction (*F* = 2.186, *p* = 0.141, ES = 0.195) was observed on the arousal score. However, a significant main effect of condition (*F* = 7.573, *p* = 0.022, 0.6 [0.1–1.2]; ES = 0.457; 1st = 2.4 ± 0.7 vs. 2.2 ± 0.7, 0.2 [−0.5–0.9]; 2nd = 3.3 ± 1.1 vs. 2.4 ± 0.5, 0.9 [0.4–1.4]; 3rd = 3.9 ± 1.1 vs. 3.1 ± 0.9, 0.8 [−0.1–1.6]) and attempt (*F* = 26.51, *p* < 0.001, ES = 0.747) was identified. The LSD post hoc comparison showed that arousal scores differed between all attempts.

#### 3.2.3. Electromyography

The RMS EMG values of the VL in the 1 RM in FC was significantly higher than those in the NC condition (*t* = 2.63, *p* = 0.0135, 57.7 [8.1–107.4] μV; ES = 0.831). Additionally, the RMS EMG values of the GS in the 1 RM in FC were significantly higher than those in the NC condition (*t* = 1.874, *p* = 0.047, 15.1 [−3.1–33.2] μV; ES = 0.593). However, no significant difference was found between conditions on the RMS EMG values of the BF during the 1 RM (*t* = 1.107, *p* = 0.149, ES = 0.35) (Table 1).

#### 3.2.4. RPE Score

**Figure 3:**
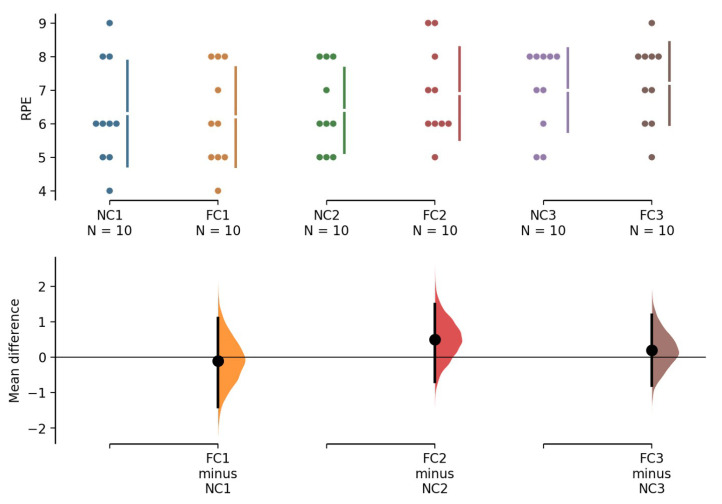
RPE values obtained at the end of attempts of 1 RM leg press test in FC and NC conditions. Gardner–Altman estimation plots showing mean difference. The 95% CI is indicated by the vertical error bar. RPE—rating of perceived exertion; RM—repetition maximal; FC—foot cooling; NC—non-cooling; CI—confidence interval.

No condition × attempt interaction (*F* = 0.403, *p* = 0.674, ES = 0.043) or main effect of condition (*F* = 0.167, *p* = 0.693, ES = 0.018) was observed on the RPE values. Only a significant main effect of attempt was found (*F* = 4.969, *p* = 0.019, ES = 0.356) (FC: 1st = 6.3 ± 1.6, 2nd = 6.4 ± 1.3, 3rd = 7.1 ± 1.2; NC: 1st = 6.2 ± 1.4, 2nd = 6.9 ± 1.3, 3rd = 7.2 ± 1.2). The LSD post hoc comparison showed that the RPE scores differed between the first and third attempts (Figure 3).

## 4. Discussion

To the best of our knowledge, this study is the first to compare the intermittent FC and NC before attempts of lower body maximal strength test in resistance-trained participants. The primary findings of this study confirmed our research hypothesis that a significant increase in 1 RM leg press performance is due to intermittent FC. In addition, under the FC condition, arousal level and RMS EMG of the VL and GS during the concentric phase of the maximal 1 RM leg press were higher than those of the NC condition. However, no difference was found in the RPE scores between the FC and NC conditions.

The cooling temperature and duration (10 °C for 2.5 min) applied in the present study avoided drastic reduction in cutaneous sensory feedback (0 °C for more than 10 min), which may blunt force production [23,24]. As demonstrated by Caminita et al. [18], squat jump height and vertical ground reaction force during squat jumping decreased after the reduction of cutaneous sensory feedback via a 15 min cooling period using an ice-water bath (approximately 0 °C) for the foot soles. However, our cooling protocol corroborates the finding that intermittent peripheral cooling at 10 °C for 2.5 min between sets of resistance exercise workouts successfully reduces the temperature of the cooling site [12,13,14] and supports the result that peripheral cooling may aid the subsequent set of resistance exercise performance [12,13,14]. A previous study reported that inter-set FC can increase the numbers of repetitions to exhaustion per set during a high-intensity (85–90% 1 RM) leg press workout [14]. Our present study further confirmed the previous findings and showed that the ergogenic effect remains to occur while a participant is engaged in 1 RM leg press exercise. This finding is similar to a previous finding that 20 min of focal knee joint cooling increases knee extension MVC in healthy adults [9]. However, our present results are not in accordance with the research conducted by Kim et al., who did not find that 20 min of focal knee joint cooling can alter isometric, concentric, and eccentric quadriceps strength at slow and fast contraction velocities in physically active participants [25]. Given that the participant populations between our present study and Kim’s study are similar, methodological differences, such as cooling sites (foot vs. knee), and cooling methods (immersion in 10 °C water for 2.5 min vs. plastic bags filled with crushed ice for 20 min), may contribute to the discrepancy. For instance, it has been demonstrated that the sensitivity of temperature sensation by cooling differs among body regions [26]. Due to the fact that the foot possesses abundant arteriovenous anastomoses [27], the heat removal and cold sensation from the foot may be higher than that from the knee, potentially causing a higher afferent stimulus [28]. Based on the results of the present study, we can conclude that FC, even at a slightly higher temperature, produces a better ergogenic effect than cooling the knee. In addition, the difference in the method of assessing strength between studies (multiple-joint exercise vs. single-joint exercise) may also account for diverse outcomes. Strategies induced the magnitude of strength performance gain in leg press (multiple-joint) tends to be more responsive than leg extension (single-joint) both acute and chronically [29,30]. Collectively, these factors may facilitate detection of significant alterations. Notably, our study highlights that even a short period (2.5 min) of intermittent cooling increases 1 RM strength in multiple-joint exercise in resistance-trained men. In addition, the magnitude of improvements ((post–pre)/pre) in 1 RM strength by FC in our study (7.1%, Table 1) was greater than those obtained using other strategies before 1 RM test in trained athletes. These strategies included incorporation of chain-loaded variable resistance into warm up (free-weight back squat: 6.1%) [31], additional eccentric loading (bench press: 3.2%) [20], plyometric depth jumps (free-weight back squat: 3.5%) [32], whole-body vibration exposure (Smith machine half squat: 4.9%) [33], and caffeine supplementation (bench press: 2.1%) [34]. The foregoing suggests that intermittent cooling of the peripheral of the active muscles provides greater benefits than those previously used strategies.

The mechanisms of improved maximal strength by peripheral cooling are not fully understood. Peripheral cooling benefits repeated resistance-based exercise performance possibly by elevating CNS arousal (as evaluated by short-term release of norepinephrine) [7], increasing peripheral motor neuron pool excitability [7,8,9], and delaying fatigue [10,11,12,13]. Previous studies have suggested that CNS arousal induced by peripheral cooling facilitates the generation of greater force by the muscle through the recruitment of additional motor neurons [7,8,9]. To the best of our knowledge, no tests of arousal levels were conducted in peripheral cooling on resistance exercise workouts. In the present study, we further investigated arousal levels using FAS. Results show that arousal scores increased significantly throughout the workout and that the participants exhibited higher arousal levels under FC than NC conditions at all time points before every attempt (Figure 2). Recent findings have suggested that elevated arousal level contributes to the ergogenic effect on the performance of more repetitions per set during a high-intensity (80% 1 RM) resistance exercise workout [35] and on performance requiring maximal motor strength over a short period [36]. The elevated arousal level observed in the present study may help explain the ergogenic effect. FC may reflect a stimulus to arouse the CNS, which may consequently allow for exerting strength to a greater extent at the following attempt.

Greater muscle force usually corresponds to higher EMG activity of the agonist’s muscles [37]. As expected, in the present study, greater 1 RM strength elicited in the FC condition was accompanied by greater RMS EMG values in VL than in NC condition. In the current study, the increased EMG values of the major muscles after peripheral cooling may be in part due to the increased CNS arousal as mentioned above [7,8,9]. In addition, peripheral cooling exerts a direct peripheral effect on permitting corresponding muscles to utilize a greater amount of motor neuron pool during MVC [9]. The current study is similar to a previous study that found significantly greater RMS EMG values in VL during a submaximal (85–90% 1 RM) leg press workout in FC compared with NC conditions [14]. We further found that FC magnified the RMS EMG values of GS while the resistance-trained participants exerted their maximal effort compared with NC, considering that the pronounced GS activation by FC has not been observed in a previous study that instructed resistance-trained participants to perform leg press exercise below 90% 1 RM [14]. Our data suggest the possibility that intermittent FC recruits additional motor units of the synergistic muscles to contract to their maximum level.

Regarding RPE scores, previous studies have shown increased numbers of repetitions with reduced RPE scores in bench press workouts after the application of inter-set palm cooling [12,13]. Inter-set peripheral cooling may possess the potential to delay fatigue or counteract some aspect of the perception of increased effort when a given amount of heavy resistance exercise workout is performed [10,11,12,13,14]. In the present study, exercise progression increased the RPE scores; however, no significant difference was found in the RPE scores between the FC and NC conditions (Figure 3). The logical inconsistency of our results with previous findings could be attributed to the work volume. In our study, the participants only performed one maximum effort in each attempt in a few seconds, and only a total of three repetitions were performed, rather than multiple sets of repetitions. Therefore, the participants may exhibit a less pronounced degree of sensations of fatigue compared with a previous study that used multiple sets of resistance exercise workouts (RPE score, 6.3–7.1 vs. 7.9–9.6) [14]. Considering arousal and EMG data of the current study, we speculate that increasing the arousal level and motor unit recruitment in corresponding muscles is a more important contributor than delaying fatigue to maximal strength improvement in response to peripheral cooling.

Our study has considerable limitations and directions for future research. First, although we measured arousal level using FAS, we did not measure brain neurophysiology, which, if measured, would provide further insights into the underlying mechanisms behind these responses. Second, the study participants are those who undergo recreational weight training. Thus, the results should be extrapolated with caution to physically inactive people, novice lifters, or professional athletes. Third, to mimic a real strength-based competition scenario and to allow standardization of the present study, three attempts and equal recovery time were given to each participant. In other research involving 1 RM testing, using gradually increased load with 2–4 min rest between lifts until participants failed to complete a lift may obtain a better 1 RM value [31]. Future studies are warranted to clarify whether FC would provide an ergogenic effect on individualized 1 RM strength testing with more attempts and rest period. Finally, the total cooling time in this study was only 7.5 min; therefore, caution should be taken when applying prolonged intermittent strength exercise between sets. Research has shown that a 30 min peripheral cooling of the knee joint and thigh muscle causes an anterior shear force on the tibia, which may have a potential risk on the ligament [38]. It is uncertain whether a risk factor would occur from the cumulative cooling effect during prolonged intermittent cooling.

## 5. Conclusions

The preliminary findings, although limited, suggest intermittent FC as a potential ergogenic role for recreationally resistance-trained participants to enhance maximal leg press strength, increase arousal levels, and recruit more motor units of the agonist’s muscles to contract to their maximum level. It can be used as an ergogenic strategy for recreational athletes performing their routine maximal strength training and may, at least in part, benefit strength-based competition events (powerlifting or Hercules). However, further studies are needed to confirm these findings in professional athletes.

## Figures and Tables

**Table 1:** 1 RM leg press and EMG activity of the selected muscle groups during the concentric phase of 1 RM load in FC and NC conditions.

Variables\Conditions	FC	NC	*t*	*p*
1 RM (kg)	195.9 ± 45	182.3 41.9	5.158	<0.001
EMG				
VL (μV)	313.2 ± 111.1	255.5 ± 74.7	2.63 *	0.0135
BF (μV)	81.8 ± 22.2	71.1 ± 26.2	1.107	0.149
GS (μV)	88.8 ± 33.4	73.8 ± 28.1	1.874 *	0.047

* Significant difference between FC and NC. RM—repetition maximum; EMG—electromyography; VL—vastus lateralis; GS—gastrocnemius; FC—foot cooling; NC—non-cooling.

## Data Availability

The data presented in this study are available upon request from the corresponding author.
